# Diagnostic and prognostic implications of testicular microlithiasis

**DOI:** 10.1080/20905998.2024.2393936

**Published:** 2024-09-03

**Authors:** Mehmet Yilmaz, Christopher Ho Chee Kong, Taymour Mostafa

**Affiliations:** a Urology, Mediclin Kraichgau-Klinik, Bad Rappenau, Germany; b Department of Surgery, Taylor’s University, School of Medicine, Subang Jaya, Malaysia; c Department of Andrology, Sexology & STIs, Cairo University, Faculty of Medicine, Cairo, Egypt

**Keywords:** Carcinoma in situ, diagnosis, male infertility, microlithiasis, tesis, ultrasonograpgy

## Abstract

Testicular microlithiasis (TML) is an uncommon condition that is discovered in the testicular parenchyma by coincidence during scrotal ultrasound examinations with a rate of 0.6–9%. Numerous medical conditions, including cryptorchidism, testicular torsion, Klinefelter syndrome, hypogonadism, varicocele, hypospadias, and infertility, have been linked to TML. Although the precise nature of the relationship between TML and infertility is still unknown, medical research has shown a correlation between abnormalities in specific sperm parameters and TML. Furthermore, studies on TML patients suggested that the results regarding reproductive hormone parameters might not be entirely consistent. If an infertile man with TML falls into one of the high-risk categories, such as having bilateral TM, atrophic testes (<12 mL), a history of undescended testis, or testicular germ cell tumors, he may be offered a testicular biopsy. The chance of getting cancer rises when TML is bilateral and there are other risk factors. Instead of doing a testicular biopsy on these individuals, a customized strategy based on the patient’s age, concurrent symptoms of testicular dysgenesis syndrome, couple fertility, desire for paternity, and ultrasound pattern is indicated for follow-up.

## KEY POINTS

- TML is an rare entity among adult men due to intratubular calcifications with highly variable incidence. TML is becoming more and more common as high-frequency transducers and US examinations for scrotal and testicular conditions become introduced.
- Men with occasionally found bilateral TML may be offered semen analysis, but reproductive hormones estimation seems unnecessary.
- TML associated with other risk factors further increase cancer development, but its likelihood risk is similar to the general population.
- In patients at risk of developing testicular cancer, observation versus testicular biopsy, an individualized approach is recommended based on the age, concurrent testicular dysgenesis syndrome, desire of paternity, fertility of the couple, and the US pattern.

## Introduction

Testicular microlithiasis (TML) is an uncommon condition that can be discovered in the testicular parenchyma by coincidence during the scrotal ultrasound (US) examination with a prevalence rate of 5.6% in patients aged 18–35 years [1]. TML can be seen unilaterally or bilaterally. This condition is known to be linked to some benign health diorders such as cryptorchidism, torsion of the testis, Klinefelter syndrome, Down syndrome, hypogonadism, varicocele, hypospadias, and infertility [1–8].

TML appears as tiny, 1–3 mm in diameter calcifications in the testis parenchyma’s seminiferous tubule lumen on US examination [5,9,10]. Histologically, these microcalcifications are composed of a hydroxyapatite core of collagen fibres and glycogen, which is responsible for the non-shadowing echoes in the testicular parenchyma on the US [10,11]. In 2015, the European Society of Urogenital Radiology proposed a classification system for TML considering three types of its counts: diffuse, limited (<5/field of view), and classic (≥5/field of view) [12].

Because TML can be benign and the microcalcification itself is not malignant, in men with isolated TML who do not have associated risk factors, the current EAU (European Urological Association) Sexual and Reproductive Health Guideline 2024 does not recommend testicular biopsy, follow-up scrotal imaging, determining tumor markers, or abdominal/pelvic computed tomography [13].

This narrative review aims to address all aspects of TML and to provide the clinicians with a comprehensive perspective.

## Relationship between TML and male infertility

### Is spermatogenesis affected?

Research has indicated a correlation between TML and abnormalities in certain sperm parameters, even though the exact nature of such relationship is still unclear [9,14]. The incidence of TML in the infertile patients was reported to vary between 0.9% and 20.1% [9]. Blockage of the seminiferous tubules can cause inflammation, which can affect the blood supply to the testes and raise the intra-seminiferous pressure leading to declined sperm quality [15]. In their study on a Chinese population of 226 TML men, Xu et al. [15] pointed out that TML was linked to poorer semen characteristics with an inverse correlation with TML sizes. Along with their study, Mahafza et al. demonstrated that the mean sperm concentration, sperm motility, and normal sperm morphology in patients with TML were; 29.6 × 10^6^/mL, 35.3%, and 44.4%, respectively, compared with 54.3 × 10^6^/mL, 50.2%, and 66.4% in the fertile controls (*p* = 0.02). These authors reported no significant variation in the semen volume between patients with or without TML [16]. Lately, Barda et al. [17] found that the sperm retrieval rate in non-obstructive azoospermia (NOA) men with TML was significantly lower compared to NOA men with no evidence of MTL (6.2% vs. 39.4%, *p* = 0.009). These authors pointed out that MTL was the solely independent predictive factor of failed sperm retrieval in the NOA group (OR = 7.4, 95% CI: 2.3, 12.2; *p* = 0.01).

Additionally, the relationship of TML with impaired sperm parameters and testicular volume has been investigated. The results of a retrospective study by D’Andrea et al. on infertile couples revealed that only low testicular volume exhibited an independent and significant association with a higher probability of detecting classic TML (OR = 0.84, 95% CI: 0.75–0.94; *p* = 0.02). Besides, in cases with classic TML, a lower mean testicular volume and a lower sperm concentration were also observed (*p* = 0.03) [18].

Along with their study on 4850 Danish males by Anvari Aria et al., bilateral TML has been reported to be linked to lower testis volume, sperm concentration, and total sperm count [19]. Similarly, another study demonstrated smaller testicular volume and lower sperm count and sperm motility in men with unexplained infertility associated with TML. Along with their study, Rassam et al. showed that impaired spermatogenesis was correlated only with the presence or absence of TML and not with its amount assessed after subdivision into <5 vs. ≥5 MTL/testis) [8]. In their series on 739 men (60 (8.1%) were diagnosed as having TML), Hiramatsu et al. [20] pointed out that the sperm concentration correlated negatively with the number of calcifications but there was no significant correlation between sperm motility and the number of calcifications.

### Would a change in the hormone parameters be expected?

Existing studies in the patients with TML show that the results regarding the reproductive hormone parameters are contradictory. Anvari et al. showed that men with bilateral TML had lower serum inhibin B and inhibin B/FSH ratio compared to those without TML, but the difference was not statistically significant. Furthermore, TML was not significantly linked to serum testosterone, FSH, luteinizing hormone (LH), or sex hormone-binding globulin [19]. Along with their study, Hiramatsu et al. reported that the mean serum LH and FSH levels were higher in men with TML compared with those without TML (4.6 vs. 3.3 mIU/mL, *p* = 0.001 for LH; 6.7 vs. 4.2 mIU/mL, *p* = 0.001 for FSH, respectively) [20]. In a cohort of men with unexplained infertility, Rassam et al. showed that the mean serum FSH levels were significantly higher in men with TML than in men without TML [*p* = 0.029] [8]. Similarly, on men of infertile couples, higher FSH serum levels were observed in cases with classical TML compared to these with limited TML [12.4 vs. 5.8 mUI/ml, *p* = 0.02] and the control group [12.4 vs. 5.3 mUI/ml, *p* = 0.009] [18] without differences in either serum total testosterone or LH levels between these groups. Therefore, more detailed, prospective randomised trials are needed regarding these points to set the association between reproductive hormone parameters and TML.

### How to manage infertile men with TML

It has been demonstrated that infertile men with TM have an 18-fold increased risk of testicular cancer compared to men without TML [21]. However, for TML men who are infertile, there is no set protocol for their follow-up. However, infertile men with TML who fall into a high-risk category may be offered a testicular biopsy, per the most recent edition of the EAU Guideline for Sexual and Reproductive Health. Testicular germ cell tumors, bilateral TM, atrophic testes (<12 mL), history of undescended testis, and spermatogenic failure are among these groups [14].

## Relationship between TML and testicular cancer

### Prevalence of TML among men with testicular cancer

The earliest examination for the prevalence of TML among men with testicular cancer was on 1982 on 92 pieces of orchiectomy with an incidence of TML to be higher in specimens containing tumours (74% vs. 16%, *p* < 0.05) [22]. In another study, the prevalence of testicular tumor in symptomatic cases with TML was 11.2% and 1% in symptomatic cases without TML (*p* < 0.001) [23]. In one more study, the prevalence was 0.68% [24] as compared to another one which was 2.4% [25]. In their recent systematic review, Aoun et al. reported a prevalence ranged from 0.9% to 20.1% [9].

However, Decastro et al. [26] suggested that testicular cancer would not develop in most men with TM (98.4%) during a 5-year follow-up. As such, an extensive screening program would only benefit men at significant risk. In this context, it would be judicious to advise patients with TM and risk factors for testicular cancer to annual physical examination and US follow-up.

### TML and testicular cancer among infertile men

Along with their systematic review on unilateral TML, Aoun et al. reported no evidence of either testicular germ cell tumor or carcinoma in situ (CIS). Otherwise, 20% of the men with bilateral TML had CIS, indicating that bilateral TML is a sign of CIS in infertile men [9]. In the largest retrospective studies on infertile men, patients with TML, Negri et al. [27] demonstrated significantly higher odds for testicular cancer with a stronger correlation with germ cell cancer in the patients with TML (*p* = 0.0015, OR 37.1) or larger calcifications not fitting the description of TML (*p* < 0.0001, OR 69.5). These findings indicated that particular care should be taken in the presence of TML or testicular calcifications and these men should be aware of the existence of higher risk of testicular cancer.

A significantly higher association between TML and testicular cancer was also observed by Sakamoto et al. as well as La Vignera et al. in their infertile patients [25,28]. However, along with four studies on a relatively smaller infertile men samples, no cases of testicular cancer were detected in cases with/without TML [29–32]. Besides, along with their meta-analysis, Barbonetti et al. pointed out that the odds ratio of detecting cancer infertile men with TML was about 18-fold higher (pooled OR: 18.11, 95% CI: 8.09, 40.55) [21].

According to De Gouveia Brazao et al. CIS was identified in 20% of their investigated patients with bilateral TML. Compared to patients without TML (0.5%) and those with unilateral TML (0%), the prevalence of CIS in infertile men with bilateral TML was significantly higher (*p* < 0.001) [33].

According to Tan et al. nearly 20% of the patients with a previous testicular germ cell tumor have TML in their contralateral testes. These patients have an increased risk ratio of 8.9 for concurrent CIS compared with patients who do not have TML [34]. In another systematic review, the infertile group showed a higher risk for testicular tumour when TML was detected on the US with OR risk 18.6 [35].

Lately, Frandsen et al. [36] carried out a cohort of 167 men who have undergone testicular biopsies because of TML and additional risk factors. This study found that men with TML and testicular atrophy are at an increased risk of germ cell neoplasia in situ. Additionally, these authors indicated that biopsies should be considered in men with a combination of infertility and bilateral TML and did not support testicular biopsies for men with TML and other risk factors.

### Management of men with TML, testicular cancer, and infertility

Surgical exploration with testicular biopsy or orchiectomy should be considered in symptomatic patients with TML and a suspicious lesion [37]. When a biopsy is recommended for reproductive purposes in TML patients, a systematic search for intratubular germ cell neoplasms (ITGCN) should be carried out. Young patients with TML and various symptoms of testicular dysgenesis syndrome may also benefit from a biopsy. Patients who have undergone an orchiectomy for testicular cancer and who have TML in the testis on the other side of observation versus biopsy are options to weigh on.

The risk in such a patient to have an ITGCN is 20% and observation is favoured when fertility issue is raised [38]. The gain of an instant biopsy is the ability to detect ITGCN, which has a 98% success rate of cure with radiotherapy alone [39]. Orchiectomy and testosterone replacement therapy can be sidestepped in these cases. Men with TML with risk factors such as bilateral TML, male infertility, cryptorchidism surgery, or testicular neoplasm should be aware about the high risk of clinically CIS, and, therefore, testis biopsy should be proposed [40].

Therefore, for TML, an individualized approach should be based on the age of the patient, presence of additional features of testicular dysgenesis, desire of paternity and the US pattern (bilateral and clustered vs. unilateral and limited) [9]. In these cases, OncoTESE and cryopreservation could be performed when after biopsy, CIS/ITGCN or cancer is encountered [40]. Table 1 presents the management of TML in different scenarios.

**Table 1.** Management of testicular microlithiasis.

Population	Management
TML with risk factors	Testicular biopsy. OncoTESE and cryopreservation could be performed when after biopsy, CIS/ITGCN or cancer is found
Symptomatic TML with suspicious lesion	Surgical exploration with testicular biopsy or orchiectomy
Young patients with TML and various symptoms of testicular dysgenesis syndrome	Biopsy
Orchiectomy for testicular cancer and who have TML in the testis on the other side	Observation vs biopsy

TML: testicular microlithiasis, TESE: testicular sperm extraction, CIS: carcinoma in situ, ITGCN: intratubular germ cell neoplasia.

### Follow-up in terms of testicular cancer risk

- TML, no risks factors (bilateral TML, male infertility linked to impaired spermatogenesis, testicular hypoplasia, cryptorchidism or history of its surgery, or testicular neoplasm), **regular testicular self-examination**.
- TML, with risk factors present, but refusing testicular biopsy – up to age 50 years, **regular scrotal US and urologist consultation**. After age 50, **regular testicular self- examination** as testicular cancer is rare after 50 years.
- TML, with risk factors present, testicular biopsy did not show CIS; **regular self-examination** [40].
- TML without testicular mass, asymptomatic with no clinical risk factors for testicular germ cell tumor– **reassurance**.
- TML without testicular mass, symptomatic, or with any risk factors for testicular germ cell tumor – **testicular self-examination and follow-up.**
- TML without testicular mass, with current or prior history of testicular germ cell tumor and received chemotherapy – **testicular self-examination and follow-up**.
- TML without testicular mass, with current or prior history of testicular germ cell tumor but not received any chemotherapy – can opt for **either testicular biopsy or no testicular biopsy.**
- If opt for testicular biopsy, in 80% of the cases, no ITGCN found, can **reassure the minimal risk of contralateral testicular germ cell tumor.**
- If opt not for biopsy, 20% risk of undiagnosed ITGCN. Half of them (i.e.10%) develop testicular germ cell tumor within 5 years. These 10% are still curable but at the point of diagnosis will require orchiectomy of remaining testis and testosterone replacement and may require adjuvant therapy [34].
- According to recent EAU guidelines, patients with TML and no testicular germ cell tumor risk factor should be encouraged to perform self-examination. Compliance is a crucial point. Testicular biopsy, follow-up scrotal US and routine use of biochemical markers are not justified [13].
